# Patents over ‘technologies’ related to how we treat, use, and modify the human body: An urgent need for greater bioethics scrutiny

**DOI:** 10.1093/medlaw/fwaf015

**Published:** 2025-08-05

**Authors:** Aisling M McMahon

**Affiliations:** School of Law and Criminology, Maynooth University, Maynooth, Ireland

**Keywords:** Patents, Licensing, Access to Medicines, Bioethics, Intellectual Property Rights

## Abstract

Under the TRIPS framework, patents must be granted in all fields of technology, including health-technologies. Patents give rightsholders significant control over patented technologies as they enable them to exclude others from using these for commercial purposes. The human body per se is not patentable. However, many technologies that relate to how we treat, use, and modify the body are patentable. For example, in Europe, patentable technologies include those that can treat the body (eg, medicines), technologies that can affect how we use elements derived from the body (eg, isolated human genes are patentable in certain contexts), and technologies that can modify (including enhance) the body (eg, neuro-technologies). Using a novel five-category taxonomy of patentable technologies related to how we treat, use, and modify the human body, this article demonstrates that such patents—and their use—can pose significant bioethical implications, focusing on implications for autonomy, dignity, and bodily integrity interests. It demonstrates that these bioethical implications are not routinely considered in European patent grant or licensing decision-making. This article challenges this. It argues that greater scrutiny is needed over these bioethical implications and over the connection that patented technologies have with how we treat, use, and modify the human body.

## I. INTRODUCTION

The human body per se is not patentable under European patent law.^1^ However, given the nature of contemporary technologies and how patent law has developed in practice, many technologies that relate to, or whose use can impact how we treat, use, and modify our bodies, are patentable. For example, under ‘European’ patent law,^2^ patentable technologies include: technologies that impact how we *treat* the human body, for example medicines; technologies that impact how we *use* certain elements derived from the human body, for example, isolated human genes derived from the body, to the extent that they disclose a specific function are patentable in Europe, and such patents can potentially impact their use including in a diagnostic context^3^; and technologies that can enable the *modification* of our human bodies, for example surgical tools.^4^ 

Focusing on the European context, this article develops a novel five-category taxonomy of patentable technologies that relate to, or whose use can impact how we treat, use, and modify the human body. It uses this taxonomy to demonstrate that rightsholders’ decisions regarding how they use patent rights over such technologies can give rise to significant potential bioethical implications.^5^ For the purposes of this article, ‘bioethical implications’ is used to refer to how patents over such technologies can impact key bioethical interests. Due to space limitations, the article focuses on the potential implications for autonomy, dignity, and bodily integrity.

More specifically, this article will examine the following aspects of such interests: (i) In the context of *autonomy*, it will be argued that: (a) how patents over health-related technologies are used is a key factor that can impact access to such technologies, which in turn can impact patient autonomy by affecting the types of healthcare treatments/interventions available to patients. (b) Relatedly, it will be shown that patents can be used in ways that impact clinical autonomy by affecting the types of healthcare interventions/treatments that clinicians/public health systems have available to provide to individual patients (or to populations more generally); (ii) Considering ‘dignity’ interests, the article acknowledges that dignity is a malleable concept.^6^ It is discussed here to refer to the implications of patents for the inherent dignity of, and respect for, individuals.^7^ For example, as will be discussed, patents over isolated human genes are seen by some as commodifying the body and hence, impacting human dignity interests.^8^ Moreover, patents over isolated genes can impact the types of genetic testing that can be conducted, which can impact what we can find out about our bodies, and this could also be seen as having implications for human dignity; (3) Finally, this article examines ‘bodily integrity’ interests, focusing on how patents over certain technologies can impact these interests by preventing unwanted physical interference with a person’s body. In this context, this article draws on Haddow,^9^ and Quigley and Ayihongbe’s work,^10^ discussed below, which highlights that certain contemporary technologies, for example medical devices, are now increasingly ‘integrated’ with the human body.^11^ It will argue that, as components of integrated medical devices are patentable (and covered by other forms of intellectual property rights (IPRs)), and as such IPRs can be used in ways that impact how (or even whether) integrated medical devices (continue to) operate, this could give rise to implications for bodily integrity interests of device users.^12^

Using the five-category taxonomy set out, and focusing on these three key bioethical interests, this article will demonstrate that such bioethical implications are not considered routinely in patent grant or use stages. Instead, once granted, a patent on an engine part is viewed the same as a patent on a lifesaving medicine,^13^ and the patent system is often agnostic to the underlying nature of a patentable technology. Moreover, whilst there are some relevant (albeit limited) exclusions from patentability at the grant stage in Europe, such exclusions are generally interpreted in a narrow and technical manner by the European Patent Office (EPO) leading to an expansive approach to patentability.^14^ Furthermore, after a patent is granted, there is no overarching legally binding framework that mandates the consideration of bioethical issues that may arise around how patents over such technologies are used (i.e., licensed or enforced). Alongside this, current legal avenues that could be used to engage with these issues, such as compulsory licensing, offer limited pathways to addressing the range of bioethical issues at stake. Against this backdrop, this article calls for a fundamental shift in how patents over such technologies are considered (both within the patent system, such as by decision-making bodies like the European Patent Office, and outside this system including within health law, bioethics and patent law communities), which would include a deeper consideration of the bioethical implications posed by patents—at both grant and use (licensing/enforcement) stages—due to how the underlying patented technologies relate to how we treat, use, or modify the human body.

In making such arguments, the article does not seek to provide an exhaustive examination of all bioethical issues that could arise in such contexts, nor is it aiming to set out a prescriptive framework for how such bioethical issues should be considered at patent grant or use stage in Europe. Its contribution is a narrower but nonetheless, significant one, namely, to make the case that the nature of a patented ‘technology’ and specifically, how a technology relates to how we treat, use, or modify the human body *matters* in the patent context. It argues that this relationship—and the attendant bioethical implications that can arise due to how patents over such technologies are granted or used—should be given greater scrutiny, including by patent law, health law and bioethics communities and key decision-making actors (such as patent offices including the EPO).

In doing so, this article fills two distinct gaps. First, there is considerable existing literature examining the ethical issues posed by patents over *specific* technologies, for example gene patents^15^; however, limited attention has been given to providing an overarching bird’s-eye examination of the bioethical issues posed by patents over a range of technologies related to the human body. This article aims to fill this gap. It seeks to draw out more generally how bioethical issues can arise in such contexts and to provoke a deeper consideration of such issues within the health law and bioethics communities. Secondly, much of the existing work on (bio)ethics and patent law focuses on questions about whether patents should be granted over specific technologies. There has been limited consideration of the role of bioethical considerations in relation to how patents are used (licensed or enforced) over such technologies.^16^ This article considers both patent grant and use stages to offer a more holistic overview and to demonstrate the range of bioethical issues that can arise.

The article is structured in five parts, including this introduction. Section II sets the foundation for the analysis by outlining the nature of patent rights. It argues that rightsholders occupy a governance function over patented technologies that gives rightsholders significant control over how such technologies are used.^17^ Building upon this, Section III sets out a novel five-category taxonomy of patentable technologies where the use of the patent can impact how we treat, use, or modify the human body. This section argues that due to the nature of the technologies within the five-categories considered, such patents can be used in ways that enable rightsholders to govern key aspects of how we treat, use, and modify the body. This, in turn, can give rise to a range of bioethical implications, including implications for autonomy, dignity, and bodily integrity interests. Section IV acknowledges that there are avenues that allow third parties to use patented technologies without the rightsholders’ permission. However, it demonstrates that these avenues are not designed to, nor can they fully address the breadth of bioethical issues at stake. Section V concludes by arguing that such bioethical issues are likely to intensify in future given the rapidly evolving nature of emerging technologies. Accordingly, it makes the case for deeper interdisciplinary scrutiny of the bioethical issues posed by patents over technologies related to the human body, and the extent to which such bioethical issues can or should be further incorporated at the patent grant and use stages.

## II. PATENTS AND RIGHTHOLDERS’ GOVERNANCE FUNCTIONS REVISITED

A patent is a type of IPR that enables rightsholders to limit others from using a protected technology for generally 20 years.^18^ A patent does not grant the rightsholder an entitlement to use this technology as this may be prohibited by national or international laws.^19^ Use of a technology may also be impacted by the existence of other patent(s) (or other forms of IPRs) in the relevant jurisdiction related to that technology, which is discussed further below. Nonetheless, provided the use is not restricted in the state where a patent is granted in, third parties who wish to (legally) use that patented technology must ordinarily seek the permission to do so (via a license) from the rightsholder(s).^20^ Licenses are generally granted by rightsholders in return for money or other forms of exchange.^21^ Rightsholders can place conditions on the use of a technology within the license, including legal clauses that prohibit certain uses.^22^ Third parties who use a patented technology without the rightsholders’ permission(s) could be challenged by the rightsholder(s) for patent infringement, and suffer penalties—such as damages—if infringement is proven. The risks of being sued for infringement—including the high costs of patent litigation^23^—can deter use of patented technologies without rightsholders’ permission.^24^ Accordingly, patents allow rightsholders considerable discretion over patented technologies: rightsholders can dictate the terms of use of the technology (including, the price), who it can be used by, and set parameters around how it is used.^25^

Furthermore, for commercial purposes, rightsholders will often seek to strategically extend the duration and breadth of their control over patented technologies. Towards the end of the patent term, rightsholders may seek to patent new uses of a technology, or new variations of the technology, such as for example, a time-release capsule version, or different delivery mechanisms changing from for example, a capsule to an intravenous injection form, to increase the duration of legal protection they hold (so-called evergreening). Rightsholders can also layer IPRs over different aspects of a technology,^26^ whereby they use (or apply for) a range of IPRs—patents, trade secrets, copyright protection, etc—over different aspects of a technology.^27^ By doing so, rightsholders can potentially create intertwined (or in some cases overlapping) legal protections, increasing their breadth of control. Multiple IPRs, held by different rightsholders, can exist over a technology, which can add complexity within the IP space^28^. In some contexts, this can make it difficult for third parties to enter a field given the multiple rightsholders they may need to negotiate with, and the uncertainties over what IPRs exist.^29^

Thus, as argued elsewhere,^30^ the control that patents (and other IPRs) bestow upon rightsholders over the use of IP-protected technologies, can enable rightsholders to occupy a governance-like function over the technology. This article uses the term ‘governance’ with reference to Braithwaite and other’s conception of ‘governance’ defined as relating to control over ‘providing, distributing, and regulating’ things.^31^ It argues that patents enable rightsholders to exercise functions akin to providing and distributing the patented technology whereby rightsholder’s decisions over how patent rights are used (and hence, how the underlying patented technologies can be used) are exercised via contractual conditions within the patent license.^32^ Using such contractual licensing conditions, rightsholders can dictate key aspects of how the patented technology is provided and distributed including to what group, and on what terms.

Such governance-like functions are demonstrated by considering the coronavirus disease-2019 (COVID-19) vaccine context. In early stages of the pandemic, limited supplies of COVID-19 vaccines were available to meet global demands. Multiple different types of IPRs applied over key aspects of these vaccines, and scaling up such vaccines for global needs would have required agreements around the sharing of such IPRs and related know-how (alongside other factors). During the pandemic, despite calls for rightsholders to share know-how and IPRs over COVID-19 vaccines to facilitate a faster scale-up of production, limited voluntary sharing of IPRs occurred in the COVID-19 vaccine context.^33^ Instead, vaccines were provided to states based on contractual agreements arranged between rightsholders and states.^34^ Patents (and other IPRs) enabled rightsholders, not the international community, to decide how vaccines were provided, and distributed, including to what state first, and on what terms, for example price, dates, etc, regardless of the global/national health needs.^35^ These contractual licensing arrangements were also used to control the follow-on distribution of COVID-19 vaccines, including the extent to which such states/regions could legally distribute unused vaccines by exportation to other states.^36^ Rightsholders’ ability to dictate the terms of distribution and provision (key features of governance) was enabled in large part by their IPRs over vaccine components.

Returning to Braithwaite and others’ definition, the third aspect of governance that they highlight relates to ‘regulation’, which they conceptualise as a ‘large subset of governance that is about steering the flow of events and behaviour’.^37^ Patents could be seen as enabling rightsholders to regulate patented technologies, because how rightsholders license a patented technology and what conditions they impose within such licenses can be used in an attempt to steer how that technology is used by others and how other technologies relying on it are developed. Rightsholders can impose clauses prohibiting certain uses of patented technologies, including where they have ethical concerns around such uses via ‘ethical licensing clauses’.^38^ Rightsholder(s) can also refuse to license patented technologies to third parties, which could lead to third parties having to develop workaround solutions, or being unable to implement an invention, thereby shaping how other technologies develop and are used.

Building upon such arguments, Section III will argue that where the underlying patented ‘technology’ relates to, or use of that technology can impact how we treat, use, or modify the human body, the governance function of the patent right can give rise to a range of bioethical implications, focusing as noted, on implications for autonomy, dignity, and bodily integrity interests. In making such arguments, this article acknowledges that patents also have an important incentivisation function for technological development within current innovation systems because patents enable rightsholders to develop an income stream over patented technologies: for example through licensing technologies. This incentivisation role of patents is a key factor that enables rightsholders to generate profits from the technology, and attract investment for emerging technologies.^39^ This article is not questioning the incentivisation role that patents play *per se—*such issues are beyond its scope—instead, the focus here is on the broader governance function of patents that exists alongside this incentivisation function, and the bioethical implications that can arise due to this which it argues requires deeper scrutiny.

Prior to examining such bioethical implications, given the institutional complexity of the European patent system, and that this article is directed also at health law and bioethics communities, a brief note is needed on the legal frameworks applicable, which will be considered below. There is no unified international patent system. An international framework setting out minimum legal standards is applicable under the World Trade Organization’s (WTO) TRIPS Agreement, as amended.^40^ This is applicable in all WTO states, including European states; however, contracting states can adopt additional obligations in certain contexts. This article focuses on the ‘European’ patent system, referring to the laws applicable under the European Patent Convention 1973, as amended (EPC). The EPC is applicable in 39 States (including all European Union (EU) States). Applicants can apply to the EPO for patents in EPC States, and if granted, the applicant obtains a bundle of national patents for the States requested.

Thus, in considering the patentability of technologies related to the human body in Europe, this article focuses on applicable EPC provisions,^41^ and where relevant, the EU’s Biotechnology Directive 1998 (applicable to biotechnological inventions).^42^ It also considers the decisions of their respective adjudicatory bodies.^43^ A unitary patent system for participating EU States recently commenced, where post-grant issues for participating States are dealt with by a new unified patent court (UPC).^44^ The article will refer to applicable legal provisions of the unitary patent system where relevant.^45^ The article focuses on the ‘European’ patent system as a case study because there are legislative provisions under the EPC and the Directive that expressly refer to ethical issues and offer avenues to engage with such (bio)ethical issues posed by patents.^46^ Given such provisions, on paper at least, it seems plausible that the ‘European’ system would engage with the bioethical implications posed by patent decision-making. Yet, in practice, this article demonstrates a marginalised approach to the engagement with such bioethical issues is evident in the European patent context.

## III. PATENTS AND TECHNOLOGIES RELATED TO THE HUMAN BODY: BIOETHICAL IMPLICATIONS

Article 5 of the Biotechnology Directive 98/44EC states:

The human body, at the various stages of its formation and development, and the simple discovery of one of its elements, including the sequence or partial sequence of a gene, cannot constitute patentable inventions.

However, despite this provision that excludes the human body itself from patentability, using a novel five-category taxonomy of patentable technologies related to the human body, this section demonstrates that patents are available in Europe over a range of technologies that relate to or whose use can impact how we treat, use, and/or modify the human body, namely: (i) technologies *derived from within the body*, such as isolated human genes, which can reveal information about the functioning of the body, or other elements derived from the body such as hormones which can have therapeuctic effects or can be used in the creation of therapeuctic products;^47^ (ii) tools/methods *used to act on the human body* whose purpose is to treat the human body or reveal information about it, such as surgical tools or elements of diagnostic processes; (iii) substances that originate *outside the body but which treat the body*, such as medicines; (iv) technologies created outside the body and intended to become *integrated with the body*, such as implantable/attachable medical devices that can be used to treat the body (such as pacemakers) and which in doing so, can also modify the body, and (v) technologies that *can be used to modify significant aspects of what it means to be human or the creation of future human life*, such as technologies that can be used for enhancement purposes (eg certain neuro-technologies) or technologies that significantly modify how we create future human beings (eg emerging assistive reproductive technologies).

This article is not suggesting that these five categories of technologies are an exhaustive categorization of all patentable technologies related to the human body where patent use can pose bioethical implications. Instead, these categories are used here as exemplars—which it is hoped others will develop further and build upon in future—to show the extent of the bioethical implications posed by patents precisely because of how the underlying patented ‘technology’ relates to how we treat, use, and/or modify the human body. Moreover, in some instances, a health-technology may span multiple aspects of these categories, for example, a medical device such as a closed-loop insulin delivery system may on the one hand, have a medical device that acts on the body, by taking measurements from the body, which it uses to calibrate blood sugar levels within the body. As a result of such readings, the system may be automated to administer specific quantities of artificial insulin—which is a substance used to treat the body—to maintain healthy levels within the body. Individuals may also be using patented technologies across a range of these technological categories at any one time. Nonetheless, the categoristaion is useful, as it allows us to examine the different types of bioethical interests that may be implicated depending on how the patented ‘technology’ relates with how we treat, use and modify the body. In doing so, it seeks to shift the focus within the patent system from a ‘technology’ centric one which looks primarily at whether a patented technology meets technological criteria of novelty, inventive step, industrial application etc, to also considering the implications of patent use, i.e., if that patent is granted, what will the potential impacts be given how the underlying patented technology relates to the human body. In particular, this categorisation seeks to encourage deeper examination of the potential implications certain patent uses may have for the broader human flourishing of the ‘technology’ user due to how that ‘technology’ relates to the human body. Ultimately, as will be discussed below, this article is not suggesting that patents should not be granted per se if bioethical implications arise, rather it argues that if such implications arise, greater consideration should be given to the need for limits on rightsholders control in such contexts, including in particular, the need for licensing conditions or patent-use conditions/guidance which would ameliorate/address such bioethical implications.

The article will now consider each of these five categories in turn. It aims to illustrate that how patents over such technologies are used by rightsholders has the potential to impact how we treat, use and modify our bodies, which can pose various bioethical implications. This analysis focuses specifically on potential bioethical implications for autonomy, dignity, and bodily integrity interests.

### A. Patentable Technologies derived from within the human body

Article 5(1) of the Directive, as noted, states that the human body *per se* is not patentable,^48^ however, Article 5(2) narrows this, it states that:

An *element isolated from the human body* or otherwise produced by means of a technical process, including the sequence or partial sequence of a gene, may constitute a patentable invention, even if the structure of that element is identical to that of a natural element.^49^

Notably, a range of different types of elements isolated or derived from the body can be patentable, for example, an early example, is considerations around the patentability of hormones derived from the human body, such as adrenaline, insulin, cortisone and sex hormones etc. Such hormones can have very important therapeutic and medicinal functions, and have been patentable in Europe, and/or led to patentable products being developed, including synethetic versions of such hormones which have important therapeuctic functions^50^. Patents over such substances derived from the body, given their therepeutic function, can impact how we treat the body. This can give rise to simliar bioethical issues as those which arise in the context of patents over medicines used the treat the body, and this is considered in category (iii) below, and hence, this aspect is not explored further in this context here. Nonetheless, other related bioethical issues arise in such contexts, around how patents over substances derived from the body can impact uses of the body (or its elements). To illustrate this, this section focuses on the patentability of isolated genes as an example where such issues also arise, and have been subject to considerable scrutiny.

In the context of isolated genes, Article 5(2) of the Directive, means that, for example, patents can be granted over isolated human genes in Europe,^51^ provided an industrial application related to the isolated gene is given within the patent application.^52^ A low threshold applies to the ‘industrial application’ requirement that focuses on disclosing a ‘technical function’ of the isolated gene. For instance, for an isolated gene to be patentable, it can be sufficient to demonstrate an educated guess or probability about its function.^53^ Patents over isolated genes, have been used in other contexts by rightsholders to argue that they have the exclusive right to isolate that gene and to subject it to diagnostic or other testing, which can impact how we use (components of) our bodies. Moreover, isolating a person’s gene allows us to examine that gene, which may reveal important information about the current or future health of that person, thereby impacting current and preventive treatment interventions. Mutations on a gene can indicate that a person has a genetic condition or that they have an increased risk of developing certain conditions. Depending on how rightsholders use patents over isolated genes or other isolated elements from the body, this can pose bioethical implications, including impacting people’s autonomy as it can affect what elements of their body can be isolated, used, and what type of testing they can undergo. Yet, patentable isolated genes are treated the same as other patentable ‘technologies’—as fungible with other patents—regardless of how patents over that gene can affect what we can do with (i.e., use) such genes and other elements of our bodies.^54^

The bioethical implications of how patents over isolated genes are used is demonstrated by considering Myriad’s use of patents over BRCA1 and BRCA2 genes in the USA in the 1990s/early 2000s.^55^ Mutations on the BRCA1 and BRCA2 genes indicate that a person has an increased risk of developing certain cancers (particularly breast and ovarian cancers). Hence, some people may seek genetic testing, and if certain genetic mutations are identified, they may seek to take preventive surgical steps to mitigate such risks arising. In the USA, Myriad claimed their patents over isolated BRCA1 and BRCA2 genes (and related methods patents) gave them the exclusive right to isolate these genes from the body. It issued cease and desist letters to other US laboratories providing genetic testing for BRCA1/BRCA2 mutations.^56^ This gave rise to several bioethical issues: Myriad became the only provider for such testing in the USA, enabling it to charge high prices, affecting who could access tests, which raised issues for patient autonomy and health equity.^57^ Their patent use strategy meant no alternative providers could offer testing, which prevented patients from obtaining second medical opinions from alternative providers if dissatisfied with Myriad’s result/test.^58^ This, in turn, raised autonomy issues for patients/clinicians. This example shows that rightsholders’ decisions over patents on isolated genes (at least in the US context as existed previously) could be used in ways which can impact our ability to subject elements of our body to certain types of testing. It also shows how rightsholders’ decisions over how they use (i.e., enforce or license) patents over isolated elements of the body can impact who can access information derived from the body using such patented technologies.^59^ Myriad’s patents were successfully challenged in the USA in 2013.^60^ Patents are not currently available over isolated genes in the USA.^61^ However, the rationale for the US decision in Myriad was based on a technical point related to the interpretation of patentability criteria in the USA—as the court found isolated genes were not patent eligible subject matter under US law. Some of the bioethical implications of patents over genes where discussed in the District Court decision in the case,^62^ however, these bioethical issues where not relevant to the core legal question at stake in the Supreme Court decision around whether isolated genes were patentable in the US context, which instead turned on a doctrinal point related to the interpretation of US patent law. Indeed, this further illuminates the disjoint between patent law and bioethics within this context, as whilst arguably a key motivation for patients, clinicans etc in challenging gene patents in the US was the implications for genetic testing, this way not the basis on which the case turned.

Moreover, patents are still available in Europe over isolated genes. Notably, for a range of reasons, Myriad’s patents were not commercialized in the same way in Europe as they were in the USA.^63^ Yet, past practice does not guarantee future approaches. There is an ongoing potential for patents (and their uses) over isolated elements of the body to give rise to bioethical implications. Such issues are heightened in the personalized medicines era, especially as we move to tailored therapies based on patients’ genetic profile, which could make patents over isolated genes or other elements of the body more important.^64^ For example, it is plausible that if new mutations on genes related to certain conditions/risk factors are identified and patented, testing for such mutations/genes in whole panel screening tests would only be possible with the rightsholders’ permissions.

Yet, as noted, Article 5(2) of the Biotechnology Directive creates a presumption of patentability of isolated genes provided other criteria are met. Moreover, in the *Relaxin* case,^65^ a challenge to the patentability of isolated genes in Europe was rejected by the EPO. Part of the *Relaxin* case focused on whether a patent over isolated genes contravened Article 53(a) EPC (the so-called morality/ordre public provision), which, as noted, states that inventions are not patentable if their commercial exploitation is against ordre public or morality.^66^ The EPO stated that it would only deny patentability under this provision to inventions where they are ‘universally regarded as outrageous’. It did not engage with the broader bioethical implications of how such patents could be used, such as their effects on genetic testing or patient/clinical autonomy.^67^ During the proceedings, it was argued that such patents were akin to patents on life and contrary to human dignity. However, these arguments were dismissed by the EPO which held that: ‘DNA as such was not life but one of the many chemical entities participating in biological processes, no offence to human dignity had occurred as the woman who donated tissue was asked for her consent and her self-determination was not affected by the exploitation of the claimed molecules.’^68^ This conveys a highly technical interpretative approach by the EPO to DNA and dignity issues. For instance, in discussing the dignity interests at stake, the EPO’s focus was solely on the dignity of the donor of the biomaterial leading to the *Relaxin* patent and whether they had given informed consent in providing their biological sample. There was no engagement with the implications of such patents for how others could access genetic testing, including impacts for future patients and their dignity or autonomy interests.^69^ Moreover, in the earlier Opposition Division decision in the case, the EPO stated that ‘the EPO is not the right institution to decide on fundamental ethical questions’ highlighting its express reluctance to engage with ethical issues posed by patents.^70^ These decisions suggest an engrained marginalization of bioethical issues at patent grant stage in Europe.

Alongside this, there are no legally binding patent licensing requirements which mandate that bioethical issues should be considered in how patents over isolated genes are licensed or enforced. There are guidelines that could be applied to address some of the bioethical issues arising. For example, the OECD guidelines on the licensing of genetic inventions (2006),^71^ updated in (2020),^72^ sets out best practices including recommendations that encourage rightsholders to license such technologies broadly for research/investigation purposes,^73^ to ensure licensor’s do not have exclusive control over the human genetic information collected,^74^ and to facilitate broad access to genetic inventions in health contexts.^75^ However, these guidelines are non-binding, and they do not mandate that bioethical issues are considered in the licensing of patents over such technologies.

In short, patents over such technologies derived from the body have the potential to be used in ways that can impact how we use elements of our body, what we can find out about how our body, including its current health and/or future genetic risk factors for disease, and this may impact what current or preventative treatment options, we are advised and choose to obtain. Yet, European patent grant and licensing/enforcement systems are largely agnostic to such issues.

### B. Patents over technologies acting on the human body

Relatedly, patents can be granted on technologies acting on the human body where the use of such technologies can reveal information about the body or be used to treat the human body. This category focuses on methods or tools used in diagnosing or treating the human body (whilst category (c) focuses on chemical substances developed outside the body used to treat the body). Article 53(c) of the European Patent Convention (EPC) states that European patents shall not be granted in respect of:

methods for treatment of the human or animal body by surgery or therapy and diagnostic methods practised on the human or animal body; this provision shall not apply to products, in particular substances or compositions, for use in any of these methods.

Thus, this provision sets out three distinct exceptions from patentability,^76^ precluding patents on: (i) methods for treatment, (ii) methods of surgery, and (iii) methods of diagnosis. However, the EPO has interpreted this provision in a narrow manner.^77^ In practice, a ‘technology’ is only excluded from patentability under this provision, if the diagnostic, surgical or therapeutic methods are practiced directly on, or in, the living human body, that is in vivo.^78^ Techniques, devices, or tools related to diagnosis, treatment, or surgery not directly practised on the body are patentable (or may have patentable elements). For example, methods of testing of bodily samples in vitro are patentable: the EPO Examining Guidelines state that: ‘diagnostic testing of blood samples is not excluded [from patentability]’.^79^

Furthermore, as confirmed by the last part of the text of Article 53(c) tools/products used as part of diagnostic or surgical processes are not excluded from patentability.^80^ Indeed, there are many examples of diagnostic, surgical, therapeutic steps/procedures, where the device and/or the substance used in the procedure are patentable, and where uses of such patents can pose bioethical implications. This section focuses on two examples, related to diagnostic testing and surgical tools, to argue that the potential effect of this exclusion is significantly narrowed in practice by the EPO’s interpretative approach in applying Article 53(c), and by the ‘exception to this exception’ contained within the provision (namely, that it does not cover the tools used in such methods). The analysis which follows highlights a range of bioethical implications that can arise as a result.^81^

First, it is useful to consider the EPO’s interpretation of the Article 53(c) methods exclusion in the diagnostic context.^82^ Here, the EPO has interpreted Article 53(c) to mean that diagnostic methods are only excluded from patentability where all steps to provide a definitive diagnosis are claimed by a patent.^83^ An interim step that is part of a diagnostic assessment is patentable. Thus, a novel technology for scanning a patient’s body and the method for this are patentable.^84^ Patents over methods or technologies used in testing bodily fluids give rightsholders the ability to control how such tests/processes are accessed and used, and by whom, which can impact access.^85^ Moreover, in the genetic testing context, these patents can be used alongside patents over the isolated genes to increase rightsholders’ breadth of control.

Liddicoat and others surveyed European laboratories to understand the ‘impact of gene-related patents on European clinical genetic laboratories’ including isolated gene patents and method patents, and found that ‘(15%) laboratories reported they had refrained from performing a test owing to a patent’.^86^ They pointed out that this does not necessarily translate into negative repercussions for patients because:

The impact on patients depends on several factors, for example: is there a satisfactory alternative medical pathway; can the patients obtain the test from a different laboratory; can funds be raised for a reasonable patent license; has the patent boosted follow-on innovation such that there is net social benefit?^87^

Whilst this is undoubtedly true, nonetheless, if patents are leading some laboratories to discontinue genetic testing, this could reduce the number of laboratories providing such testing. If there is no, or limited, competition in the market, this could increase rightsholders’ ability to charge a high price, especially where there is no alternative medical pathway. A nuanced consideration is needed here, as patent grant *per se* can also have important incentive effects in the current health innovation system; however, this evidence suggests that how such patents are used can potentially impact patient/healthcare providers’ personal/clinical autonomy by impacting the tests available, their price, and accessibility.

There is also evidence of rightsholders more actively enforcing patents in the non-invasive pre-natal testing (NIPT) context in Europe than in the single gene testing context.^88^ Hawkins’ empirical work has found that compared to the single gene testing, ‘[t]hose involved in the development and delivery of NIPT are more aware of patents, and balance the costs and benefits of greater engagement or compliance’.^89^ Moreover, she argued that greater attention towards compliance (and hence, not using the technologies without a patent license) could lead to a reduction of NIPT options available, as some entities may simply opt not to offer a test, and others may opt not to offer a test where they cannot negotiate a license on favourable terms.^90^ Furthermore, Hawkins argued that in the public sector negotiating of IP rights:

…adds an extra layer of complexity .... Moreover, while commercial providers who provide testing … can pass on increased costs to the consumer, where testing is to be provided in the public sector, when multiplying those costs across the population, those increased margins can make a test uneconomic.^91^

Thus, patents have the potential to be used by rightsholders in ways that impact the availability and types of NIPT testing available, which can, in turn, pose bioethical implications, such as impacting the tests available to a pregnant person, which could impact their autonomy; impacting the availability of different (test) providers and thus individuals’ ability to obtain a second opinion from a different provider; and impacting clinical diagnostic options that can be provided. It is possible for States to adopt IP licensing guidelines for NIPT (or other contexts), which could address or ameliorate such bioethical issues, including around access to NIPT technologies. However, there is limited evidence that this is likely in European States or at a regional/supranational level, at the time of writing. Moreover, beyond NIPT technologies, similar issues could arise in other contexts due to rightsholders’ discretion over how patents are used, which does not generally account for the underlying technology’s connection with the human body or potential bioethical implications that can arise due to patent use. Thus, the NIPT context as discussed here offers an exemplar to highlight the broader impact patents may pose for access and delivery of diagnostic testing services in Europe.

Secondly, aside from how the scope or potential effect of these exclusions can be narrowed by interpretation in practice, another practical limitation around how such exclusions may address bioethical issues is that Article 53(c) does not cover tools used in surgery, diagnostic, or therapeutic processes. Thus, whilst methods of surgery are not patentable in Europe under Article 53(c), products or tools used in surgery are patentable. Such patents can impact surgeons’ ability to perform surgery in particular ways. For example, in the computer aided surgery context, certain technologies that guide surgical instruments, simulate or model surgical procedures and surgical robotic devices are patentable.^92^ The number of new patents applied for over such technologies has grown significantly in Europe since 2005.^93^ The impact of patents on surgical tools over how surgery is performed is demonstrated by considering the *da Vinci* surgical system.^94^ This surgical robotic device system was launched in 1999, with multiple IPRs applying over different elements of the system.^95^ On average, each da Vinci surgical system reportedly costs over €1.5 million, with additional parts estimated as costing over €1000 per operation.^96^ A divide has developed between which hospitals can afford such devices, and those that cannot. Patents are not the only factor contributing to or driving such costs, but they are an important factor because patents can limit alternative providers’ ability to develop and/or provide similar technologies. As key patents have started to expire in the surgical robotics context, it has been reported that other companies have begun developing surgical robotic devices (often) at lower prices.^97^

How such patents are used over these technologies can affect clinicians’ autonomy, impacting how they can treat the human body. It also impacts patients’ ability to obtain certain surgical procedures by impacting the tools available for conducting surgery—impacting our ability to modify bodies and, in some cases, such patent use may potentially impact healthcare outcomes. Like other contexts, patent grant and licensing systems are agnostic to the connection between the patented technology and the human body. There are no legally binding licensing requirements over how rightsholders license patents over surgical tools in Europe, such as accessibility or price stipulations.

### C. Patentable technologies outside the body which treat our human bodies

Thirdly, technologies developed outside the human body which are used to treat the body are also patentable. As noted, methods of treatment practised within the human body are not patentable; however, Article 53(c) does not exclude substances or tools used to treat the body. This category focuses on the patentability of substances used in the treatment of the human body. The term ‘treatment’ is used expansively here to include medicines and preventive (or so-called prophylactic) treatments used to prevent disease, such as vaccines. It also could include contraceptive medicinal products, such as oral contraceptive pills etc, used as a means to seek to prevent pregnancy. There is considerable literature on the impacts of patents on access to medicines, which this article does not duplicate.^98^ Instead, it seeks to focus on how the technologies subject to patent—such as elements of medicines or vaccines—relate to how we treat, use or modify the body. Viewed through this lens and examined alongside the other technological categories, it argues that because of how patentable technologies relate to the human body, including how we ‘treat’ the body, the use of patent rights can have significant bioethical implications which warrant greater consideration as part of this wider discussion.

Considering first, preventive treatments, the COVID-19 vaccine context discussed above shows that how patents (and other IPRs) over elements of vaccines are used can impact vaccine access, including in health emergencies. During the pandemic, a significant gap developed between low- and middle-income countries (LMICs) and high-income countries’ (HICs) access to COVID-19 vaccines—this gap has decreased but is still evident.^99^ Without global vaccine coverage during the pandemic, the risk of new COVID-19 strains arising was heightened. Such strains could have been more severe or resistant to vaccines. Patents were a key factor that impeded the expansion of COVID-19 vaccine production during the pandemic, particularly in LMICs, and arguably, how patents/IPRs impacted global vaccine access was contrary to effective pandemic management.

Such issues are not confined to health emergencies. For example, vaccines against the human papillomavirus virus (HPV)—the leading global cause of cervical cancer—are (or previously were) inaccessible within many LMICs. A key factor impacting LMICs’ access to such vaccines is how IPRs can enable rightsholders to control how HPV vaccines are distributed globally.^100^ The HPV vaccine market is dominated by a small number of providers. For example, a 2015 study highlighted that two companies dominated the patenting field for HPV vaccines. It found that the number of patent claims over these vaccines, and the lack of certainty around the scope of such claims, led to uncertainty for new manufacturers developing biosimilar versions.^101^

A systemic issue is that many LMICs often depend on charitable donations via aid programmes, companies’ donations, or other external sources to access vaccines.^102^ Accordingly, as LMICs are often unable to pay prices that HICs can offer, LMICs can struggle to access vaccines where there are short supplies globally. For example, Tanzania had a designated HPV vaccination programme supported and run via GAVI, but could not provide vaccines during the early stages of its HPV vaccine campaign because GAVI could not procure enough vaccines for LMICs—largely due to demand from HICs for such HPV vaccines.^103^ At that time, HPV vaccines reportedly cost approximately $250 in the USA, whereas GAVI sought a discounted price for the vaccine of approximately $3–5/vaccine in GAVI eligible countries.^104^ It is unsurprising that HICs obtained access to such vaccines first given these price differentials particularly, as many rightsholders are for-profit companies, which often operate under (and have a legal duty to consider) a shareholder primacy model, which is typically understood to mean they must aim to maximize shareholder value (where value tends to focus on shareholder profits).^105^

Lack of vaccine access in LMICs has significant health implications—for example, in the HPV context, in 2020, 90 per cent of cases/deaths from cervical cancer globally were in LMICs, and 60 per cent of deaths occurred in countries that had not introduced HPV vaccination.^106^ Screening, testing, and treatments for cervical cancers are limited in LMICs compared to HICs.^107^ Hence, if there is a limited vaccine supply globally, the public health rationale would suggest vaccines should go first to LMICs. However, in practice, rightsholders (often commercial entities) exercise a governance function enabled by such IPRs over vaccines, and they dictate how vaccines are distributed (including to which countries) and on what terms—such decisions are often based on states purchasing powers not necessarily health needs.

Similar issues are evident around the pneumococcal vaccine, which protects against pneumonia. Over 700,000 children under 5 years of age die annually from pneumonia; such deaths are largely preventable.^108^ Until recently, two companies, Pfizer and GSK, held the majority of patents for the pneumococcal vaccine, maintaining high costs with limited access to such vaccines in LMICs.^109^ Following civil society campaigns,^110^ GAVI negotiated with rightsholders for vaccines to cost less than 5 per cent of public costs.^111^ However, these agreements are only applicable in GAVI eligible States. They do not apply to many middle-income states. Moreover, there are no legally binding restrictions on how IPRs over such vaccines are used. As will be discussed in Section IV, States can issue compulsory licenses on patents, including on patents over elements of vaccines, but compulsory licenses are often seen as exceptional or as a ‘last resort’ in practice.^112^ IPRs enable rightsholders to dictate the distribution of vaccines where there are limited supplies, and the bioethical issues posed by rightsholders’ control over elements of vaccines is simply not considered within patent law.

The impact of patents on how we treat the body is also prevalent in the access to medicines context. A widely discussed example relates to the impact of patents on access to anti-retroviral treatments (ARVs) during the HIV/AIDs crisis in the 1990s. Patents were used to retain high prices for ARVs and limit generic production in LMICs, as a result many people, particularly in Africa, were unable to access ARVs. The Doha Declaration was signed against this backdrop.^113^ This Declaration clarified that measures could be taken under TRIPS by states to support public health, including the use of TRIPS flexibilities such as compulsory licensing provisions to alleviate the impacts IPRs could have on access to health.^114^ Broader access to generic ARVs was eventually delivered globally, but millions died before this.^115^ The tensions around patents and access to health continue to this day. These issues are not confined to LMICs, instead, they are increasingly affecting HICs. For example, a WHO report in 2021 demonstrated that one in every two people who required insulin did not have sufficient access globally.^116^ Studies from 2019 to 2022, showed that three main companies—Sanofi, Novo Nordisk, Eli Lilly—controlled 99 per cent of the global insulin market by value and 96 per cent by volume.^117^ Moreover, when key patents over insulin expire, patents are often applied for new uses or formulations of these.^118^ Patents also apply over materials needed to make insulin products and devices to administer insulin and can be used in ways that can pose additional access barriers, including barriers for producers of generic/biosimilar versions.^119^ Importantly, the existence of patents *per se* here is not the issue, rather, it is how these rights enable rightsholders relatively unfettered discretion to govern uses (including making, etc) of the underlying technology, which can impact access. The impacts of patents on access to medicines/therapies in HICs are also evident for cancer immunotherapies,^120^ obesity drugs,^121^ and a range of other medicines, where high costs enabled by IPRs mean these are inaccessible to many. Moreover, where such medicines are provided by public health systems at high costs, opportunity costs arise because it may mean other medicines cannot be provided.

These examples illustrate the potential health implications posed by how patents over such medicines/vaccines are used, which in turn can have bioethical implications. For instance, these examples show that how patents are used can impact State autonomy over health/vaccination programmes as vaccines/medicines may be unaffordable for States, and biosimilar/generic versions are deterred by how patents operate. Broader questions around health equity also arise, as countries that can pay more often access vaccines/medicines first, regardless of health needs. Moreover, how such patents are used can impact people’s autonomy over what vaccines and/or medicines etc they can obtain. Even though many such (patented) technologies are often integral to how we treat our bodies (and to how people may seek to prevent disease in the case of vaccines, or how people may seek to prevent pregnancy in the case of contraceptive medicinal products), there are no binding legal requirements that differentiate how patents can/should be used over vaccines/medicines and other patentable technologies despite these bioethical implications.

### D. Patentable technologies that are integrated with the body

Fourthly, patents can be granted over technologies external to, but which are ‘integrated’ with the body,^122^ for example implantable medical devices, such as pacemakers. How patents over such technologies are used, can impact access, supply, and use of such devices, with knock-on effects for how we treat the body, as in many cases integrated technologies aim to restore/maintain our bodies’ healthy functioning. Indeed, as Haddow argues, due to technological developments, people’s bodies are increasingly ‘everyday cyborgs’—‘“hybrid[s] of machine and organism”’.^123^ Take a pacemaker, because this is intended to be implanted into the body, one could argue that pacemakers become part of our bodies.^124^ Typically, multiple IPRs (including multiple patents) exist over elements of a pacemaker, for example patents on hardware forming the pacemaker, patents, or copyright on certain aspects of the software encoded in the device, etc.^125^ How IPRs over such implantable medical devices are used can affect the development of, and access to, such devices. For example, rightsholders’ decisions over patents on component parts of a device will affect whether these parts can be used to develop a pacemaker (as licenses over such patents are needed for their use in this process), and this can impact costs and the terms of access for such devices.

The impact rightsholders’ discretion can have on users’ ability to use such devices is demonstrated, for example, by considering the Argus II retinal implant system (bionic eyes) produced by Second Sight. This device was implanted in over 350 people globally, enabling people who had lost or had limited sight to have a form of vision.^126^ However, Second Sight ran into financial difficulties and discontinued this implant in 2019. It stopped producing upgrades on software used to operate the device. People with the implanted devices lost their ‘vision’, thereby losing a key bodily function.^127^ Moreover, to have such devices fitted, device users had to undergo surgery modifying their bodies so the implant could operate. This surgery was invasive and entailed risks. Despite this, after undergoing such surgery, the implanted device subsequently became obsolete, and users had limited legal recourse. Leaving obsolete devices in the body entailed risks of medical complications. Yet, removing the device was not without risks as this would also involve an invasive procedure which would be potentially painful for device users, and expensive.^128^ This scenario resulted due to financial difficulties experienced by Second Sight and was not attributable to the use of IPRs *per se*. However, patents (and other IPRs) over devices could be used in a way that would lead to a similar result: for instance, a rightsholder could, in theory, decide to no longer repair or upgrade elements of, or the software needed for, medical devices to function.^129^ Rightsholders could also legally use their IPRs to try to block third parties offering upgrades or repairs on such devices, including by using patents, or other IP, infringement actions. This could leave individuals with an implanted device that impacts how their body functions rendered ineffective.^130^ Such scenarios are entirely legally possible and demonstrate that the discretion rightsholders have poses bioethical implications due to the nature of the underlying patented technology (the implanted medical device) and its connection with the human body, and more specifically, the blurring of the body and technology in such contexts.^131^ It is conceded here that rightsholders may be dissuaded from strategies that result in such technologies being rendered ineffective (at least without offering due warning to users), in some cases, due to the potential for reputational fallout and public backlash that could arise. Nonetheless, much is left to the discretion of rightsholders in such contexts due to the IPRs applicable, and how such rights can be used. In short, how such patents (and other IPRs) are used can impact the accessibility and usability of integrated medical devices, thereby impacting individual users’ autonomy in terms of whether they can obtain such devices in the first place.

Furthermore, and more radically, in such scenarios, patents could be seen as giving rise to implications for bodily integrity interests. In terms of ‘bodily integrity’ and contemporary technologies, Herring and Wall have previously argued that:

…we can also appreciate why Judge Greve in Price v UK viewed the act of depriving a person who lacked “ordinary legs and arms” access to her battery charger for her wheelchair as interference with her bodily integrity.^132^ A more radical implication of the idea of “leaky bodies” is that external objects that share a functional unity with the body can form part of the body for the purposes of the right to bodily integrity.^133^

Herring and Wall’s conception of bodily integrity in this contemporary context is one they argued related ‘to the integration of the self and the rest of the objective world’ where the notion of the human body is construed as a fluid one and understood in a subjective sense.^134^ Applying this conception and such arguments to the patentable ‘integrated’ medical device contexts—and focusing here on technologies created externally to the body that are intended to be assimilated with the body—if patents are used in ways that constrain how the integrated technologies that become parts of the human body operate after implantation, this could be seen as amounting to an interference with our bodies. Hence, such patents (and other IPRs)—and how these are used—could amount to an interference with bodily integrity—because in such cases it is the rightsholders not device user that dictates how such patents (or other IPRs) are used including where such patent/IP use impacts the operation of the device (and hence the user’s body).

Yet, under the current European patent law system, there are limited avenues to address these issues. At grant stage, as noted, the human body *per se* is not patentable (Article 5, Biotechnology Directive). However, integrated medical devices comprises different component parts that form a device. That overall device—and these parts—are developed in a technical manner outside the body. Hence, for patent law purposes, which typically assesses patentability at the point of technological development, such technologies are not part of the human body *per se*, and patents would not be precluded from patentability by Article 5(1). One might seek to argue that given the impact of rightsholders’ decisions on how they use patents over such technologies, the commercial exploitation of the technology is against ordre public/morality and patentability should be contrary to the general morality provision (Article 53(a) EPC). However, as noted, the general morality provision is typically only applied in rare circumstances to deny patents in practice.^135^ It is primarily used to deny patents over technologies whose use would be abhorrent. Patents over integrated medical devices would be highly unlikely to be contrary to the morality provision as currently interpreted. Furthermore, the key issue from a bioethical perspective is not related to the grant of the patent *per se*, but rather with how that right once granted can be used by rightsholders. Yet, there are no overarching provisions mandating that in using such patents (i.e., in licensing or enforcing patents) the rightsholders needs to consider the potential bioethical implications of these decisions for how the underlying integrated medical device technologies can operate.

### E. Patentable technologies that can enhance the human body in significant ways, or alter the creation of future human life

Fifthly, patents are available over technologies that are intended to be used in ways which could ‘enhance’ the human body in significant ways,^136^ and technologies that alter the creation of human beings and future human life (for example, in assisted human reproductive contexts). Such issues are considered together here because in both contexts, the underlying technologies could fundamentally alter how we interact as humans with each other and the world around us.^137^ More radically, in the human reproductive context, depending on how technologies and regulatory systems develop, emerging techniques such as genome editing (if other regulatory systems were to allow use of such technologies in future—which remains uncertain) could impact human genetic identities with knock-on implications for future generations.^138^ Accordingly, this section demonstrates that where elements of such technologies (including, underlying processes used by these) are patentable, how key aspects of the use of such technologies could be controlled via patents creates significant bioethical implications. As will be discussed, such technologies may overlap with technologies in other categories, but here the focus is on uses for enhancement and/or reproductive purposes. This is because although patents give rightsholders significant governance functions in all contexts, however, in this context, due to the implications of the underlying technologies for how we interact with the world, and for the future of humanity more generally, the bioethical issues around rightsholders’ control are arguably heightened.

First, patents are available on technologies that have the potential to enhance human bodies in significant ways, including enhancing our cognitive abilities. Relevant emerging technologies in this context could have multiple uses: having the potential to offer therapeutic functions; and the potential to be used by or on healthy bodies to enhance the body (e.g., if we consider CRISPR gene editing technologies). Technologies that enhance/modify our bodies could fall within several other categories considered above, for example, implantable devices with therapeutic/enhancement purposes could also be categorised as integrated technologies under the taxonomy proposed. However, the bioethical issues posed by patents over technologies with enhancement potential are discussed separately here because of the distinct bioethical issues around control of enhancement type technologies. To demonstrate this, we can consider brain computer interface (BCI) technologies—BCIs have multiple potential uses, including therapeutic functions to give people control over bodily functions or implantable/attachable prosthetic limbs/devices. However, BCIs could also be developed for enhancement purposes. In this context, it is useful to consider the Neuralink device. Neuralink is the size of a coin, and when implanted in the skull, it connects the brain with an external electronic device. The Neuralink website states its aim or mission as being for Neuralink to be used in future to ‘eventually expand how we experience the world’ and to ‘unlock human potential tomorrow’,^139^ and the founder Musk previously indicated his aim for such technologies to enable humans to communicate via computer interfaces and directly link human brains with such interfaces.^140^ This implies an intention to use such technologies to enhance how we communicate with each other and technologies around us. There are uncertainties around whether such aims are scientifically possible; however, it is at least plausible that BCIs could be developed, which have cognitive enhancement capabilities.

Patents already apply over components of BCI technologies^141^; however, where the patented technology is intended for enhancement purposes, arguably this raises a distinct set of bioethical issues: Patents over such technologies give rightsholders, many of whom are commercial actors/entities, significant control over the development of technologies, which could shape fundamental aspects of what it means to be human, and of the world we live in. Regulatory safeguards against certain uses of neurotechnology for human enhancement may be imposed by scientific regulations. However, such regulations may allow neuro-technologies for enhancement purposes in future. Indeed, some authors have argued—albeit contentiously—that there may even be a moral duty to enhance our bodies.^142^ Where such enhancement technologies are patented, rightsholders could exert significant gatekeeping functions over access to (including by setting the price of such devices), development, and use of such technologies. Given that the use of such technologies could fundamentally alter key elements of the ‘human’ experience, it is questionable if it is ethically appropriate for rightsholders to have governance functions that shape the development, access, and uses of these technologies, particularly as rightsholders’ profit maximization goals may not align with societal needs.

Secondly, patents are available over aspects of emerging assisted reproductive technologies (ARTs) related to the creation of human life. These technologies are considered within this category, because of their potential to be used in assisted human reproductive (AHR) contexts which can have significant benefits for individuals or couples, but also (depending on the technologies) could have, or could have when combined with other technologies, for example gene-editing, the potential to be used to alter, including in ways which intend to enhance future generations. Some emerging reproductive technologies (or related technologies) are subject to multiple patents, which give rightsholders (often commercial entities) significant control, and this arguably raises significant ethical questions.

For example, Cyranoski, Contreras and Carrington highlight the increasing proliferation of patents related to emerging ARTs today contrasting this to the early development of in vitro fertilization(IVF) technologies.^143^ They note the growing numbers of patents related to emerging in vitro gametogenesis (IVG) technologies—a procedure that involves development of gametes (egg and sperm) from undifferentiated human cells, such as adult skin cells, which offers reproductive potential for couples/individuals experiencing infertility.^144^ Greely predicts that IVG could account for up to 90 per cent of human reproduction in future.^145^

Certain aspects of IVG technologies would likely be excluded from patentability in Europe; for example, patents are not available over human beings or human embryos in Europe.^146^ Furthermore, Article 6(2)(c) of the Biotechnology Directive excludes patents over ‘uses of human embryos for industrial or commercial purposes’, which has been interpreted to exclude patents over technologies that involve the destruction of a human embryo at any stage in the development of that invention.^147^ However, IVG involves the creation of gametes, and this poses new questions. For instance, depending on the technique used to create such gametes, if this did not involve destruction of an embryo, it would not necessarily be excluded under Article 6(2)(c) in Europe. Furthermore, as discussed above, there is an exclusion on patents over methods of treatment (Article 53(c) EPC), and if IVG was construed as a therapy, methods of such therapy are excluded from patentability in Europe. However, even if that were the case, other aspects of IVG technologies could still be patentable in Europe. As noted, tools and substances (such as medicines, etc) used in treatment are patentable. Hence, even if certain methods of IVG fell under the therapeutic methods exclusion in Europe, the tools/medicines (or other chemical substances) used as part of the IVG process could still be patentable. Moreover, whilst the general morality provision in Europe could be invoked to argue the commercial exploitation of such ‘inventions’ is against *ordre public*/morality, however, as noted, this provision is typically only used in rare and exceptional contexts.^148^ Thus, it is unlikely the EPO would deny patents related to IVG or other future ARTs on this basis, especially as the technology has many beneficial uses for society.

On the societal implications of the growing patent numbers for the delivery of IVG, Cyranoski, Contreras and Carrington, argue:

…the administration of IVG may shift from thousands of independent clinics around the world to a handful of corporate providers or their licensees. The implications of this shift are difficult to predict at this early stage. Yet while much has been written about the need to move slowly to ensure that IVG risks are minimized, we must also ensure that its fruits are broadly distributed without exacerbating existing global reproductive disparities.^149^

Such points are highly significant for the argument raised here. Put simply, given the increasing commercialisation (including via patents) of such ARTs, rightsholders’ decisions over patents related to such technologies could impact an individual’s reproductive autonomy, whereby rightsholders could act as gatekeepers over the development of emerging ARTs. Their decisions on how they use such patents could also impact the price of obtaining ARTs and related services, with implications for the accessibility of AHR, the use of specific ARTs in healthcare systems, and which countries or people can avail of these technologies. Due to how such technologies can impact how we create new human life and can also potentially impact key aspects of the nature of future human life, the role and breadth of rightsholder discretion over these technologies warrants much deeper scrutiny.

## IV. BIOETHICS AND AVENUES WITHIN PATENT LAW TO INTERVENE WITH RIGHTSHOLDERS DISCRETION POST GRANT

Section III demonstrated that patents—and their use—over technologies in the categories discussed, can pose bioethical implications, including impacting, autonomy, dignity, and bodily integrity interests, given the connection of such technologies with the human body. There are certain exclusions from patentability in Europe; however, as discussed, such provisions are often limited in how they could be used to address the range of bioethical issues posed by contemporary technologies. There are no general legal provisions that require bioethical issues to be considered as part of how patented technologies are licensed or used. For completeness, this section considers three main post (patent) grant avenues, which allow individuals/third parties to legally use a patented technology without the rightsholders’ permission. In some instances, such avenues could be used to ameliorate some of the specific bioethical issues discussed. However, this section shows that such avenues are not designed to, nor do they, address the breadth of bioethical issues at stake.

First, acts done or use of a patented technology for private and non-commercial use is not an infringement of a patent (so-called personal use exemption). The scope of this exemption depends on the national legal framework applicable, although for European unitary patents, this exemption is now enshrined at a supranational level for participating States under Article 27(a) of the Agreement on a Unified Patent Court Agreement (UPCA).^150^ Due to the recent nature of the UPCA, there is limited jurisprudence on how this provision will operate in practice under the UPCA. Thus, it is useful to consider an example of the operation of this provision at the national level, for instance, in the UK. Section 60(5) of the Patents Act 1977 states that: ‘An act which, apart from this subsection, would constitute an infringement of a patent for an invention shall not do so if—(a) it is done privately and for purposes which are not commercial.’ However, this exemption is not designed to address bioethical issues posed by patents, and for a range of reasons is ineffective in doing so. First, in terms of access issues, the personal use exemption may assist where a person is able to make a patented technology for their own personal use. However, given the complex nature of many technologies related to the body, including medicines, vaccines, and medical devices, most individuals are unlikely to be able to do so. Even if they were able to do so, making such technologies for personal use could give rise to safety concerns. It is likely that, at best, an individual with no access to the required patentable technology could seek a third party to make or provide that technology for the individual to use. However, the act of making a patented technology by a third party (without permission from the rightsholder) is (typically) not covered by the personal-use exemption. There are examples of individuals using the personal-use exemption to import generic versions of medicines for personal use—generics may be possible to legally produce in other jurisdictions, for example, where the medicine is not patented in that other jurisdiction.^151^ Nonetheless, the use of this avenue would be dependent on an individual user having sufficient knowledge around how to source that medicine, and the user being able to pay for the generic version and arrange importation.^152^ Such acts could also fall foul of customs laws, depending on the State.^153^ Another limitation of the personal use exemption in addressing bioethical issues is that whilst it may assist for medicines, it is unlikely to address access issues where a medical procedure on a person’s body is required to implant the technology with the body (e.g. for implantable medical devices), or where medical expertise is needed to develop repair parts/services for implantable medical device technologies, or for diagnostic or therapeutic techniques. In short, this exemption is primarily a defense for use of patentable technologies by individual users; it does not offer an avenue to curtail or limit rightsholders’ discretion generally over how they use patents over technologies related to how we treat, use, or modify the human body due to the bioethical issues that can arise.

Secondly, the experimental use/research exemption allows researchers to use patented technologies for research purposes without being considered to have infringed the patent right. The scope of this exemption differs across national jurisdictions but is often interpreted narrowly.^154^ The UK context, is useful to consider as a example here, as it has relevant case law around the scope of the research exemption under UK law, and as the UK remains a party to the EPC, despite its recent withdrawal from the EU. Indeed, it remains a key patent jurisdiction within the EPO system. In the UK context, uses of a patented technology that have an intended commercial purpose, including developing a new technology with a commercial aim, are likely not to be covered by this exemption.^155^ Thus, this avenue cannot be used to enable an entity to produce a generic or biosimilar version of a patented technology, as this would be unlikely to fall within the definition of ‘research’. Depending on the national context, it may allow a researcher to use a patented technology for research purposes if they were researching an invention for non-commercial purposes, but if they intended to develop a new product using this research, it would be unlikely to fall within the exemption.

Thirdly, under certain circumstances, as confirmed by Art 31 of the TRIPS Agreement, a compulsory license (CL) can be granted by the government to allow a third party to produce/use a patented technology, subject to adequate remuneration being paid to the rightsholder(s).^156^ The Doha Declaration (2001) confirmed WTO States abilty to use TRIPS flexiblities including the freedom ‘to determine the grounds upon which CLs are granted’. However, in practice, there are several limitations to the use of CL to address potential bioethical implications posed by use of patents over technologies related to the body. For example, a CL can only be applied for in respect of a patent (not for other IPRs).^157^ Furthermore, CLs are granted at a national level, and the practical steps needed in each State for a CL are governed by national laws. In some countries, the process for applying for a CL can be bureaucratic and burdensome. For example, States can include additional requirements for a CL other than the minimum requirements set out in Art 31 of the TRIPS Agrement or Art 5 of the Paris Agreement 1883, as amended, making a CL difficult to obtain in practice.^158^ Moreover, some States, may fear trade sanctions, or backlash from industry, if they issue a CL.^159^ Alongside such issues, at a practical level, multiple different patents may exist over a technology, it may be difficult for those seeking a CL to determine which patents apply, and depending on the national system for the grant of a CL, CLs may need to be applied for separately for each patent applicable. Thus, whilst the CL system is useful in certain contexts to address access issues, for example, around single-molecule drugs that can be reversed engineered. However, the CL system is not designed to address the broader bioethical issues posed by how rightsholders exercise their discretion over technologies related to the body, more generally.

Accordingly, while these avenues allow access to, and use of, a patented technology without the rightsholders’ permission in certain contexts, they only apply in limited circumstances. These avenues are by no means intended to, nor are they appropriate to address the breadth of bioethical issues at stake.

## V. CONCLUSION

This article has argued that patents over technologies that relate to how we treat, use, and modify the body can pose significant bioethical implications, including the potential to affect autonomy, dignity, and bodily integrity interests. Such issues are not fully grappled with by current patent grant and use systems in Europe. Moreover, we are on the cusp of an age where it will be possible (or even routine) to use patentable technologies to enhance the human body, and where key elements of emerging ARTs may be patentable. This increases the potential bioethical issues that may arise around patents over such technologies. Despite this, currently, barring limited exceptions, the relationship that patented technologies have with how we treat, use or modify the human body is not considered within patent grant and use decision-making systems more generally. Rightsholders occupy a largely unfettered governance role over such technologies. Against this backdrop, this article has made the case that it is vital there is a deeper scrutiny of the bioethical implications posed by patent grant and use over technologies related to the human body. In short, we must reframe the focus within patent decision-making to consider the technologies’ relationship with the body in a way that radically acknowledges, but also challenges, the extent of rightsholders’ governance role over how we treat, use and modify our bodies.

## Footnotes

1 art 5, Directive 98/44/EC of the European Parliament and of the Council of 6 July 1998 on the legal protection of biotechnological inventions (‘Biotechnology Directive’).

2 This article focuses on ‘European’ patent law referring to laws applicable in European Patent Convention 1973, as amended (EPC), contracting states.

3 art 5(2) Biotechnology Directive 98/44EC. Isolation of genes is needed for certain types of genetic testing. Thus, patents over such genes, can potentially impact the types of genetic testing that can be conducted. This is discussed further below. Other elements derived from the body are also patentable, for example, various hormones derived from the human body which have important therapeuctic functions are patentable. Patents over such hormones could be seen as impacting how we use such isolated elements derived from the body, with knock on implications for how we treat the body, given their therapeuctic effects in many cases. For a discussion of patents and therapeutic hormones, see: Graham Dutfield, That Highest Design of Purest Gold: A Critical History of the Pharmaceutcial Industry 1880–2020 (World Scientific Publishing, 2020), chapter 6 ‘Making Hormones’ at 219–245.

4 Some technologies may impact more than one aspect of how we treat, use, or modify the body. For example, a medical device may impact how we treat and modify the body. The fact that technologies fall within more than one category does not change the argument related to the bioethical impacts of patents over such technologies due to their link with the human body.

5 Technologies can have multiple patents over them, or more than one rightsholder. The article uses ‘rightsholders’ throughout.

6 The malleable nature of dignity is discussed in: Christopher McCrudden, ‘Human Dignity and Judicial Interpretation of Human Rights’ (2008) 19 Eur J Intl Law 655; Jeremy Waldron, ‘Dignity and Rank’ (2007) 48 Eur J Sociol 201; Aurora Plomer, ‘Human Dignity and Patents’ in Christophe Geiger (ed), *Research Handbook on Human Rights and Intellectual Property* (Edward Elgar 2015).

7 Roberto Andorno, ‘Human Dignity and Human Rights as a Common Ground for a Global Bioethics’ (2009) 34 J Med Philos 223.

8 DB Resnik, ‘DNA Patents and Human Dignity’ (2001) 29 J Law Med Ethics 152.

9 This draws on the conception of medical devices ‘integrated’ with the body/persons, in: Muireann Quigley and Semande Ayihongbe, ‘Everyday Cyborgs: On Integrated Persons and Integrated Goods’ (2018) 26 Med Law Rev 276. Gill Haddow and others, ‘Cyborgs in the Everyday: Masculinity and Biosensing Prostate Cancer’ (2015) 24 Sci Culture 484; Gill Haddow, *Embodiment and Everyday Cyborgs: Technologies that Alter Subjectivity* (Manchester University Press 2021).

10 Quigley and Ayihongbe ibid.

11 See also, as discussed below: Jonathan Herring and Jesse Wall, “The Nature and Significance of the Right to Bodily Integrity” (2017) 76 Cambridge Law J 566.

12 See also: Quigley and Ayihongbe (n 9).

13 Graham Dutfield, *That High Design of Purest Gold: A Critical History of the Pharmaceutical Industry, 1880–2020* (World Scientific 2020).

14 For instance, in the context of the morality provision, see: Aisling McMahon, ‘Institutions, Interpretive Communities, and Legacy in Decision-making: A Case Study of Patents, Morality, and Biotechnological Inventions’ in Edward S Dove and Niamh Nic Shuibhne (eds), *Law and Legacy in Medical Jurisprudence: Essays in Honour of Graeme Laurie* (CUP 2022*)*; Siva Thambisetty, ‘The Institutional Nature of the Patent System: Implications for Bioethical Decision-Making’ in C Lenk, N Hoppe and R Arnaudo (eds), *The Ethics and Law of Intellectual Property* (Routledge 2016). More generally, on the interpretation of the exclusions from patentability by the EPO, see: Sigrid Sterckx and Julian Cockbain, *Exclusions from Patentability: How Far Has the European Patent Office Eroded Boundaries?* (CUP 2012).

15 Graeme Laurie, ‘Patents, Patients and Consent: Exploring the Interface between Regulation and Innovation Regimes’ in H Somsen (ed), *The Regulatory Challenge of Biotechnology* (Edward Elgar 2007); Graeme Laurie, ‘Patenting and the Human Body’ in I Grubb, A Laing and J McHale, *Principles of Medical Law* (OUP 2010); Lori B Andrews and Jordan Paradise, ‘Patents: The Need for Bioethics Scrutiny and Legal Change’ (2005) 5 Yale J Health Policy Law Ethics 403; For a related discussion of the narratives of human persona within patent law which considers broader bioethical concerns including around human dignity, see: Hyo Yoon Kang, “Identifying John Moore Narratives of persona in patent law relating to inventions of human origin” in P., Atkinson, and Greenslade, H. eds. *New Genetics, New Social Formations*. (London, New York: Routledge, 2007).

16 For discussion in the context of health-technologies more generally, see: Aisling McMahon, ‘Accounting for Ethical Considerations in the Licensing of Patented Biotechnologies and Health-Related Technologies: A Justification” in Naomi Hawkins (ed), *Patenting Biotechnological Innovation: Eligibility, Ethics and Public Interest* (Edward Elgar 2022) 163.

17 Aisling McMahon ‘Biotechnology, Health and Patents as Private Governance Tools: The Good, the Bad and the Potential for Ugly?’ (2020) 18 IPQ 161; Aisling McMahon ‘Global Equitable Access to Vaccines, Medicines and Diagnostics for Covid-19: The Role of Patents as Private Governance’ (2020) 47 J Med Ethics 142; Duncan Matthews and others, ‘The Role of Patents and Licensing in the Governance of Human Genome Editing: A White Paper’ Queen Mary Law Research Paper No. 364/2021 <https://ssrn.com/abstract=3896308> accessed 21 January 2024.

18 On the length of patent term protection—Art 33 TRIPS Agreement 1994, as amended; art 63, EPC.

19 For example, a rightsholder may have a patent over abortion related technology. However, if abortion is legally prohibited, they cannot use that technology.

20 There are limited exceptions, discussed below. See also, Matthews and others (n 17).

21 For eg, a cross-licensing arrangement.

22 CJ Guerrini and others, ‘The Rise of the Ethical License’ (2017) 35 Nat Biotechnol 22; McMahon (n 16).

23 Katrin Cremers and others, ‘Patent litigation in Europe’ (2017) 44 Eur J Law Econ 1; For UK, see: Christian Helmers and Luke McDonagh, ‘Patent Litigation in England and Wales and the Issue-based Approach to Costs’ (2013) 32 CJQ 369.

24 Patents may not deter all users—Hawkins highlights there can be ‘wilful blindness’: Naomi Hawkins, ‘The Impact of Human Gene Patents on Genetic Testing in the United Kingdom’ (2011) 13 Genet Med 320.

25 McMahon (n 17).

26 Siva Thambisetty and others, ‘Addressing Vaccine Inequity during the COVID-19 Pandemic: The TRIPS Intellectual Property Waiver and Beyond’ (2022) 81 Cambridge Law J 384.

27 IPRs such as copyright, trade-secrets do not need to be applied for/registered. For a discussion of various aspects of overlapping intellectual property rights, more generally, see: Neil Wilkof, Shamnad Basheer and Irene Calboli (eds), *Overlapping Intellectual Property Rights* (2nd Edition, Oxford University Press 2023); Estelle Derclaye & Matthias Leistner, *Intellectual Property Overlaps: A European Perspective* (Hart Bloomsbury, 2011).

28 The classic problem created by patent thickets was outlined in: Michael Heller & Rebecca S. Eisenberg, “Can Patents Deter Innovation? The Anticommons in Biomedical Research” (1998) 280 Science 698. Available at: https://scholarship.law.columbia.edu/faculty_scholarship/1158 (last accessed 26 May 2025).

29 In the COVID-19 mRNA vaccine context, see: Mario Gaviria and Burcu Kilic, ‘A Network Analysis of COVID-19 mRNA Vaccine Patents’ (2021) 39 Nat Biotechnol 546.

30 McMahon (n 17). Matthews and others (n 9), Jacob S Sherkow, ‘Patent Protection for CRISPR: An ELSI Review’ (2017) 4 J Law Biosci 535.

31 John Braithwaite and others, ‘Can Regulation and Governance Make a Difference?’ (2007) *Regulation and Governance* 1-7.

32 McMahon (n 17). For a critical analysis of the role of contracts in such contemporary contexts, see: TT Arvind and Aisling McMahon, ‘Contracts, Relationality and Unilateral Governance’ (Working Paper, on file with author).

33 Indeed, the main voluntary mechanism for this WHO’s COVID-19 Technology Access Pool (C-TAP), had limited uptake in the vaccine context during the height of the pandemic, see discussion in: Susi Geiger and Aisling McMahon, “Analysis of the institutional landscape and proliferation of proposals for global vaccine equity for COVID-19: too many cooks or too many recipes?” (2023) 49(8) J Med Ethics 583. For an overview of the C-TAP initiative, see: <https://www.who.int/initiatives/covid-19-technology-access-pool> accessed 26 May 2025.

34 Alison Slade and Naomi Hawkins, ‘Intellectual Property Rights and Advance Purchase Agreements in a Crisis’ (2023) IPQ 1.

35 Mark Anderson, Naomi Hawkins and Alison Slade, ‘Contracts and Intellectual Property Rights in Translational R&D: Furthering Safeguards in the Public Interest’ (2024) 42 Nat Biotechnol 179.

36 ibid; ‘Covid Vaccines Produced in Africa Are Being Exported to Europe’ *New York Times* (New York, 16 August 2021) <https://www.nytimes.com/2021/08/16/business/johnson-johnson-vaccine-africa-exported-europe.html> accessed 27 January 2024.

37 Braithwaite and others (n 30); Julia Black, ‘Critical Reflections on Regulation’ (2002) 27 Aust J Legal Philos 1; Julia Black, ‘Constitutionalising Self-Regulation.’ (1996) 59 Modern Law Rev 24.

38 McMahon (n 17); Matthews and others (n 17).

39 There are debates around the extent to which patents are an efficient incentivization mechanisms within health innovation systems, and it is important to acknowledge that patents are seen as poor incentivisation tools in some areas (e.g. in the vaccine context). Moreover, inequalities/opportunity costs can arise in such contexts, however, such issues are outside the scope of this article. On the misalignment between patent incentives and vaccine development, see: Ana S. Rutschman, *The Intellectual Property of Vaccines: Takeaways from Recent Infectious Disease Outbreaks*, (2020) 118 Mich. L. Rev. Online 170.

40 Agreement on Trade-Related Aspects of Intellectual Property Rights (TRIPS) 1994, as amended.

41 The EPC is applicable to all types of inventions.

42 Following its adoption, the European Patent Organization adopted the Directive as supplementary interpretation for the EPC, and thus, provisions of the Directive guide the EPO in how it interprets the patentability of biotechnological inventions. See: Antonia Bakardjieva-Engelbrekt, ‘Institutional and Jurisdictional Aspects of Stem Cell Patenting in Europe (EC and EPO): Tensions and Prospects’ in Aurora Plomer and Paul Torremans (eds) *Embryonic Stem Cell Patents in Europe: European Law and Ethics* (OUP, 2009) 227-269.

43 These are the boards of the EPO, and the Court of Justice of the European Union (CJEU).

44 The unitary patent system sets up a route for applicants to apply for a unitary patent in participating EU States. This system is only open to EU Member States who choose to participate. See: Karen Walsh, ‘Institutional Coexistence: The Necessity of Judicial Dialogue and Cooperation in the UPC’ in Duncan Matthews & Paul Torremans, *European Patent Law: The Unified Patent Court and the European Patent Convention*. (de Gruyter, 2023). There is a transitional period within which applicants can opt out of the UPC’s remit.

45 However, as the unified patent court was recently established there is limited relevant jurisprudence to date. This is correct at the time of writing, 12th August 2024.

46 These provisions are discussed in detail in section III. They include the general morality provision under Art 53(a)EPC which excludes patents over inventions—applicable to all technological fields—whose commercial exploitation is against ordre public/morality (see also: Art 6, Biotechnology Directive); the exclusion against patents on methods of treatment, surgery or diagnosis of the body (Art 53(c), EPC). A range of other jurisdictions have simliar morality provisions, for a discussion see: World Intellectual Property Organization, ‘Certain Aspects of National/Regional Patent Laws’, <www.wipo.int/export/sites/www/scp/en/national_laws/exclusions.pdf> accessed 1 June 2025. See also discussion in: Matthews and others, (n 17) section 2.3.2.

47 A range of elements derived from the human body, for example hormones such as adrenaline, insulin and cortisone have important therapeutic uses, and have been patentable in various forms. As Dutfield notes: “Today we are living with the legacy of the hormones era⋖once hormones emerged as products, patent granting offices and courts were generally quite favourably disposed towards hormone-related patent claims. In countries allowing chemicals to be patented, hormones were inherently patentable whether as synthetic chemicals or as preparations extracted from body tissue or fluid. On the basis of similar rationales, genes, microbes, plants and animals are now patentable too⋖” p. 241, Graham Dutfield, That High Design of Purest Gold: A Critical History of the Pharmaceutical Industry 1880–2020 (World Scientific Publishing 2020), for full discussion see chapter 6 & chapter 9. see also (n 3) above.

48 See also discussion in: Justine Pila, ‘Intellectual property rights and detached human body parts’ (2014) 40 *Journal of Medical Ethics* 27-32.

49 Emphasis added.

50 On the history of patents over hormones, and related technologies, see: Dutfield, (n 3), chapter 6.

51 As noted, the Directive has been adopted as supplementary interpretation by the European Patent Organization and its influence is evident on how the EPO applies the EPC, see: Implementing Regulations to the Convention on the Grant of European Patents of 5 October 1973, Part II, Chapter V, Rule 26; Guidelines for Examination at the European Patent Office (EPO), Chapter II, Rule 5.2.

52 art 5(3), Biotechnology Directive.

53 In the UK—Human Genome Sciences v Eli Lilly [2011] UKSC 51 at [123]; EPO decisions: T 1329/04, para 6, 11, T 0640/04, para 6, T 0898/05, paras 8, 21, 27 and 31, T 1452/06, para 6, T 1165/06 para 25.

54 TT Arvind and Aisling McMahon, ‘Commodification, Control and the Contractualisation of the Human Body’ in Elodie Bertrand, Marie-Xavière Catto, Alicia Mornington (eds) The Limits of the Market (Mare & Martin 2020).

55 Naomi Hawkins, ‘A red herring: Invalidity of human gene sequence patents’ (2016) 38 European Intellectual Property Review 83.

56 Richard E Gold and Julia Carbone, ‘Myriad Genetics: In the Eye of the Policy Storm’ (2010) 12 Genet Med S39-70.

57 Robert Cook Deegan and A Niehaus, ‘After Myriad: Genetic Testing in the Wake of Recent Supreme Court Decisions about Gene Patents" (2014) 2 Curr Genet Med Rep 223.

58 Hawkins (n 45); Arvind and McMahon (n 51); Aisling McMahon, ‘Gene Patenting and the Marginalisation of Ethical Issues,” (2019) Eur Intellect Property Rev 608.

59 Morton Ebb Juul Nielsen, Nana Cecille Halmsted Kongsholm and Jens Schovsbo, ‘Property and Human Genetic Information’ (2019) 10 J Commun Genet 95; Mateo Aboy and others, ‘How Does Emerging Patent Case Law in the US and Europe Affect Precision Medicine?’ (2019) 37 Nat Biotechnol 1118.

60 569 U.S. 576 (2013); See: Jorge Contreras, *The Genome Defense: Inside the Epic Legal Battle to Determine Who Owns Your DNA* (Algonquin Books 2021).

61 There were previously proposals under consideration in the USA to change this, under the proposed Patent-Eligibility Restoration Act of 2023, which was not adopted. See: Michael Rosen, ‘Where Does Patent Reform Stand as 2023 Comes to a Close?’ <https://www.aei.org/technology-and-innovation/where-does-patent-reform-stand-as-2023-comes-to-a-close/> accessed 16 February 2024; See also discussion in: Robert Cook Deegan et al, “Sorry you asked? Mayo, Myriad, and the battles over patent-eligibility” (2024) Journal of Law and the Biosciences lsae010, https://doi.org/10.1093/jlb/lsae010; The proposal was recently re-introduced as the proposed Patent Eligibility Restoration Act 2025. This 2025 proposal has been changed in some respects, and for example, isolated genes per se are not seen as patentable subject matter under this proposal. Nonetheless, if adopted it could have broader relevance in future to the area and field more generally in terms of exceptions to patentability. See discussion in: Gene Quinn, “Patent Eligibility Reform Returns to the Hill: PERA 2025 Explained” IP Watchdog (1 May 2025) https://ipwatchdog.com/2025/05/01/patent-eligibility-reform-returns-hill-pera-2025-explained/id=188610/

62 For a discussion of the different types of narratives in the arguments around Myriad’s gene patents in the US context, including how these were discussed in judicial decisions on the case, see: Jorge L. Contreras, “Narratives of Gene Patenting” (January 6, 2016). (2016) 43 Florida State University Law Review 1133, available at SSRN: https://ssrn.com/abstract=2485681

63 Different patent cultures may explain some differences: Shobita Parthasarthy, *Patent Politics: Life Forms, Markets, and the Public Interest in the United States and Europe* (University of Chicago Press 2017), chapters 1, 5; There were European challenges to the BRCA1 and BRCA2 patents, see: Case number T 1213/05-3.3.04.

64 See generally: McMahon (n 55); Arvind & McMahon (n 51). For a discussion of impact of patents in the non-invasive prenatal testing context: see: Naomi Hawkins, “Patents and non-invasive prenatal testing: Is there cause for concern?” (2021) 47(5) Science and Public Policy 655-667; Naomi Hawkins et al, “The continuing saga of patents and non-invasive prenatal testing” (2019) 39(6) Prenat Diagn, 441-447.

65 Case T 0272/95 (*Relaxin/Howard Florey Institute*) of 23 October 2002.

66 art 53(a) EPC, art 6(1) Biotechnology Directive 98/44EC.

67 McMahon (n 55).

68 Case T 0272/95 (n 61).

69 Arvind and McMahon (n 51).

70 Decision of the opposition division (Relaxin/Howard Florey Institute) 8 December 1994.

71 OECD, ‘Guidelines for Licensing of Genetic Inventions’ <https://www.oecd.org/science/emerging-tech/36198812.pdf> accessed 6 November 2023; Diane Nicol, ‘Cooperative Intellectual Property in Biotechnology’ (2006) 4 SCRIPTed 136.

72 OECD, ‘Recommendation of the Council on OECD Legal Instruments the Licensing of Genetic Inventions’ <http://registronacional.go.cr/Recomendacion%20licenciamiento%20OCDE%20OECD-LEGAL-0342.pdf> accessed 6 November 2023.

73 ibid 2.1.

74 ibid 1.5.

75 ibid 2A–2D.

76 It also applies to animal bodies that is beyond the scope of this article.

77 See: Sterckx and Cockbain (n 14) ch 5.

78 Case T1197/02 [2007] EPOR9; See also: G 0001/04 (Diagnostic methods) 16 December 2005; G 0001/07 (Treatment by surgery/MEDI-PHYSICS) 15 February 2010.

79 European Patent Office, Guidelines for Examination, 4.2.1 <https://new.epo.org/en/legal/guidelines-epc/2023/g_ii_4_2_1.html> accessed 6 November 2023.

80 art 53(c) states: ‘this provision shall not apply to products, in particular substances or compositions, for use in any of these methods’.

81 This interpretive approach is arguably more problematic as the EPO’s stated rationale for the exclusion is ‘based on socio-ethical and public health considerations’. See: Sterckx and Cockbain (n 14) 157 and 5.1.

82 This section focuses on the application of art 53(c) EPC in the diagnostics methods context because due to limits on space it is not possible to also offer an analysis of how the exemption applies for methods of surgery and therapy. However, similar arguments can be made in the therapy/surgery contexts.

83 Cygnus/Diagnostic Methods, G 1/04 [2006] OJ EPO 334, 352. See: Bruker/Non-invasive measurement, T 385/86 [1988] EPOR 357. See discussion in Lionel Bently and others, *Intellectual Property Law* (6th edn, OUP 2024) 6.2.3.

84 Abbe Brown and others, *Contemporary Intellectual Property: Law and Policy* (5th edn, OUP 2018) 482.

85 Maarten van der Heijden, ‘Preliminary Literature Review on Patents and Diagnostics’ Summer 2017 - Review conducted for Médecins Sans Frontières <https://msfaccess.org/sites/default/files/2020-05/Diagnostics%20Lit%20Review%20Final.pdf> accessed 6 November 2023.

86 John Liddicoat and others, ‘Continental Drift? Do European Clinical Genetic Testing Laboratories Have a Patent Problem?’ (2019) 27 Eur J Hum Genet 997.

87 John Liddicoat and others, ‘Continental Drift? Do European Clinical Genetic Testing Laboratories Have a Patent Problem?’ (2019) 27 Eur J Hum Genet 997.

88 Naomi Hawkins and others, ‘The Continuing Saga of Patents and Non‐invasive Prenatal Testing’ (2019) 39 Prenatal Diagnosis 441.

89 Naomi Hawkins, “Patents and Non-invasive Prenatal Testing: Is There Cause for Concern?” (2020) 47(5) Sci Public Policy 655, 656.

90 ibid 664.

91 ibid 664.

92 International Patent Classification (IPC) code for ‘Computer-aided surgery and manipulators or robots specially adapted for use in surgery’ (A61B 34/00) as discussed in GJE Intellectual Property, Computer-Aided Surgery: Patent Strategy in Europe (2022) <https://www.gje.com/wp-content/uploads/2022/05/Computer-Aided-Surgery-Patent-Strategy-in-Europe.pdf> accessed 6 November 2023.

93 ibid 4. There is an exclusion against patents on computer programs *as such* in Europe; however, patents may be available over computer programs used to achieve certain technical functions.

94 Bec Crew, ‘Worth the Cost? A Closer Look at the da Vinci Robot’s Impact on Prostate Cancer Surgery’ (2020) 580 Nature S5.

95 Rafael Andrés Clavijo Rodríguez and René Javier Sotelo Noguera, ‘New Horizons in Robotic Surgery: DaVinci Begins to Compete’ (2021) 30 Urol Colomb e153.

96 Benedict O’Donnell, ‘Robot-assisted Surgery Moves Towards the Mass Market’ <https://ec.europa.eu/research-and-innovation/en/horizon-magazine/robot-assisted-surgery-moves-towards-mass-market> accessed 6 November 2023; Michela Tindera, ‘Robot Wars: $60B Intuitive Surgical Dominated Its Market For 20 Years. Now Rivals Like Alphabet Are Moving In’ (*Forbes*, 14 February 2019) <https://www.forbes.com/sites/michelatindera/2019/02/14/intuitive-surgical-stock-robot-surgery-da-vinci-alphabet-jnj-ceo-gary-guthart/?sh=458efd5ea37b> accessed 6 November 2023.

97 Eisaku Nitta, ‘Surgical Robots Face Cheaper Rivals as Key Patents Expire’ (*Nikkei Asia*, 11 April 2021) <https://asia.nikkei.com/Business/Health-Care/Surgical-robots-face-cheaper-rivals-as-key-patents-expire> accessed 6 November 2023; N Mayor and others, ‘Past, Present and Future of Surgical Robotics’ (2022) 13 Trends Urol Men Health 7; Barry A O’Reilly, ‘Patents Running Out: Time to Take Stock of Robotic Surgery’ (2014) 25 Int Urogynecol J 711.

98 For eg, see: Sigrid Sterckx, ‘Patents and Access to Drugs in Developing Countries: An Ethical Analysis’ in Michael J Selgelid and Thomas Poggee (eds), *Health Rights* (Routledge 2010); Duncan Matthews, ‘The Right to Health and Patents’ in Christophe Geiger (ed), *Research Handbook on Human Rights and Intellectual Property* (Edward Elgar Publishing 2015); Emmanuel Kolawole Oke, *Patents, Human Rights, and Access to Medicines* (CUP 2022), Brigette Tenni and others, ‘What Is the Impact of Intellectual Property Rules on Access to Medicines? A Systematic Review’ (2022) 18 Global Health 40.

99 <https://data.undp.org/vaccine-equity/> accessed 6 November 2023. For a discussion on the role of IPRs in the COVID-19 context and implications for the next pandemic, see: Haochen Sun and Madhavi Sunder (eds), *Intellectual Property, COVID-19, and the Next Pandemic: Diagnosing Problems, Developing Cures* (Cambridge University Press, 2024).

100 Chaevia Clendinen and others, ‘Manufacturing Costs of HPV Vaccines for Developing Countries’ (2016) 34 Vaccine 5984; Mario Songane and Volker Grossmann, ‘The Patent Buyout Price for Human Papilloma Virus (HPV) Vaccine and the Ratio of R&D Costs to the Patent Value’ (2021) 16 PLoS One e0244722.

101 Subhashini Chandrasekharan and others, ‘Intellectual Property Rights and Challenges for Development of Affordable Human Papillomavirus, Rotavirus and Pneumococcal Vaccines: Patent Landscaping and Perspectives of Developing Country Vaccine Manufacturers’ (2015) 33 Vaccine 6366.

102 Swathi Padmanabhan and others, ‘Intellectual Property, Technology Transfer and Manufacture of Low-cost HPV Vaccines in India’ (2010) 28 Nat Biotechnol 671; Sara Eve Crager, ‘Improving Global Access to New Vaccines: Intellectual Property, Technology Transfer, and Regulatory Pathways’ 2014) 104 Am J Public Health S414.

103 Stephanie Nolan, ‘Money, Sex and Rumors: Tanzania Faces Challenges to Protect Girls From HPV’ *New York Times* (New York, 23 May 2023) <https://www.nytimes.com/2023/05/23/health/hpv-vaccine-tanzania.html> accessed 6 November 2023.

104 ibid.

105 See discussion in: SS Kuo and B Means ‘Corporate Social Responsibility after Disaster’ (2012) 89 Washington Univ Law Rev 973, 995; P Ireland ‘Shareholder Primacy and the Distribution of Wealth’ (2015) 68 Modern Law Rev 49; M Yan ‘Corporate Social Responsibility versus Shareholder Value Maximization: through the Lens of Hard and Soft Law’ (2019) 40 Northwestern J Int Law Business 47; H Hansmann and R Kraakman ‘The End of History for Corporate Law’ (2001) 89 Georgetown Law J 439. For a discussion of a broader emerging enlightened shareholder approach, see: A Keay ‘Tackling the Issue of the Corporate Objective: An Analysis of the United Kingdom’s “Enlightened Shareholder Value Approach”’(2007) 29 Sydney Law Rev 577: M Min and others, ‘Should Pharmaceutical Companies Engage in Corporate Social Responsibility?’ (2017) 36 J Manage Dev 58; Aisling McMahon and Edana Richardson, ‘Patents, Healthcare and Engaged Shareholders: A Pathway to Encourage Socially Responsible Patent Use?” (2022) 42 Legal Stud 271.

106 UNICEF, ‘Closing the Gap: UNICEF Bolsters Country Efforts to Increase HPV Vaccination’ <https://www.unicef.org/supply/stories/closing-gap-unicef-bolsters-country-efforts-increase-hpv-vaccination> accessed 6 November 2023.

107 ibid.

108 UNICEF, Pneumonia (December 2022) <https://data.unicef.org/topic/child-health/pneumonia/#:∼:text=A%20child%20dies%20of%20pneumonia,of%20these%20deaths%20are%20preventable> accessed 6 November 2023.

109 Médecins San Frontiers, ‘MSF Challenges Pfizer’s Monopoly on Lifesaving Pneumonia Vaccine in Korea’ <https://msfaccess.org/msf-challenges-pfizers-monopoly-lifesaving-pneumonia-vaccine-korea-0> accessed 6 November 2023.

110 See: Médecins Sans Frontiers, ‘A Fair Shot for Vaccine Affordability: Understanding and Addressing the Effects of Patents on Access to Newer Vaccines’ <https://www.msfaccess.org/sites/default/files/2018-06/VAC_report_A%20Fair%20Shot%20for%20Vaccine%20Affordability_ENG_2017.pdf> accessed 6 November 2023.

111 Gavi, ‘Pneumococcal Vaccine Price Drops for Third Year Running’ <https://www.gavi.org/news/media-room/pneumococcal-vaccine-price-drops-third-year-running> accessed 6 November 2023; Gavi, ‘Pneumococcal Vaccine Support’ <https://www.gavi.org/types-support/vaccine-support/pneumococcal> accessed 6 November 2023.

112 Ellen ‘t Hoen and others, ‘Medicine Procurement and the Use of Flexibilities in the Agreement on Trade-related Aspects of Intellectual Property Rights 2001-2016’ (2018) 96 Bull World Health Organisation 185.

113 World Trade Organization, Doha Ministerial Declaration on TRIPS and Public Health, 2001. WT/MIN(01)/DEC/2.

114 See: Carols Correa and Duncan Matthews, ‘Discussion Paper: The Doha Declaration Ten Years on and Its Impact on Access to Medicines and the Right to Health’ (UNDP, December 2001).

115 Report for the International Intellectual Property Institute, ‘Patent Protections and Access to HIV/AIDS Pharmaceuticals in Sub-Saharan Africa’ <https://www.wipo.int/export/sites/www/about-ip/en/studies/pdf/iipi_hiv.pdf> accessed 16 February 2024.

116 World Health Organisation, ‘Keeping the 100-Year-Old Promise: Making Insulin Access Universal’ <https://www.who.int/publications/i/item/9789240039100> accessed 6 November 2023; Ryan Knox, ‘Insulin Insulated: Barriers to Competition and Affordability in the United States Insulin Market’ (2020) 7 J Law Biosci. 1–25 (January-June 2020), lsaa061.

117 David Beran and others, ‘A Perspective on Global Access to Insulin: A Descriptive Study of the Market, Trade Flows and Prices’ (2019) 36 Diabet Med 726: D Beran and others, ‘Constraints and Challenges in Access to Insulin: A Global Perspective’ (2016) 4 Lancet Diabetes Endocrinol 275.

118 Warren A Kaplan, and Reed F Beall, ‘The Global Intellectual Property Ecosystem for Insulin and Its Public Health Implications: An Observational Study’ (2017) 10 J Pharm Policy Pract.

119 A Olsen and others, ‘Patents and Regulatory Exclusivities on FDA-approved Insulin Products: A Longitudinal Database Study, 1986–2019’ (2023) 20 PLoS Med e1004309; J Luo and Aaron S Kesselheim, ‘Evolution of Insulin Patents and Market Exclusivities in the USA’ (2015) 3 Lancet Diabetes Endocrinol 835.

120 European Cancer Patient Coalition, ‘CAR-T Therapy: White Paper’ <https://ecpc.org/wp-content/uploads/2022/11/White-Paper-CAR-T-therapy.pdf> accessed 16 February 2024; Luis Gil Abinader and Jorge L Contreras, ‘The Patentability of Genetic Therapies: CAR-T and Medical Treatment Exclusions around the World’ (2019) 34 Am Univ Int Law Rev 705, 727.

121 For a discussion of US patents over obesity related medicines see: R Alhiary and others, ‘Patents and Regulatory Exclusivities on GLP-1 Receptor Agonists’ (2023) 330 JAMA 650; Jacob Levi and others, ‘Estimated Minimum Prices and Lowest Available National Prices for Antiobesity Medications: Improving Affordability and Access to Treatment’ (2023) 31 Obesity 1270.

122 In terms of ‘integrated’ technologies, this article draws on the conception of ‘integrated’ in this context, as discussed in: Quigley and Ayihongbe (n 9) that discussed the integration of persons and goods in the context of such medical device technologies.

123 Haddow (n 9).

124 On embodiment of the body and devices, see Haddow (n 9); see also Quigley and Ayihongbe, (n 9), Muireann Quigley and Laura Downey, ‘Integrating the Biological and the Technological: Time to Move Beyond Law’s Binaries?’ in Edward Dove and Niamh Nic Shuibhne (eds), *Law and Legacy in Medical Jurisprudence: Essays in Honour of Graeme Laurie* (CUP 2021) 279.

125 For a more detailed overview examining the range of IPRs that may be applicable over such medical devices, and potential bioethical issues that can arise in this specific context, see: Aisling M McMahon and Opeyemi I Kolawole, ‘Intellectual Property Rights over “Integrated” Medical Devices: The Potential Health Impacts and Bioethical Implications of Rightsholders’ Control’ (2025) 33 Med Law Rev. 1–25.

126 Eliza Strictland and Mark Harris ‘Their Bionic Eyes are Now Obsolete and Unsupported’ (Spectrum, 15 February 2022) <https://spectrum.ieee.org/bionic-eye-obsolete> accessed 11 November 2023.

127 Anders Sandberg, ‘Your eyes will be discontinued: what are the long-term responsibilities for implants?’ (*Oxford Practical Ethics Blog*, 18 February 2022) <http://blog.practicalethics.ox.ac.uk/2022/02/your-eyes-will-be-discontinued-what-are-the-long-term-responsibilities-for-implants/> accessed 11 November 2023; Liam Drew, ‘Like Taking Away a Part of Myself’ - Life after a Neural Implant Trial’ (2020) 26 Nature Med 1154; Liam Drew, ‘Abandoned: The Human Cost of Neurotechnology Failure’ (*Nature*, December 2022).

128 Strictland and Harris (n 122).

129 For eg, as part of a planned obsolescence strategy, see: Quigley and Ayihongbe (n 9).

130 Contractual or other clauses could be specified in relevant agreements to add safeguards. However, given the impact on users and the power imbalances at stake, arguably broader ethical obligations should be considered for example, obligations/conditions considered at ethics approval stages in the clinical trial’s context. For a discussion on the need for obligations to clinical trial participant’s post-trial, see: Odile C Van Stuijvenberg and others, ‘Leaving Users in the Dark: A Call to Define Responsibilities toward Users of Neural Implanted Devices’ (2022) 13 AJOB Neurosci 233; Ishan Dasgupta and others, ‘What Happens after a Neural Implant Study? Neuroethics Expert Workshop on Post-Trial Obligations’ (2024) 17 Neuroethics 1–14.

131 On the blurring of bodies and technologies: Christoph Bublitz, ‘The Body of Law: Boundaries, Extensions, and the Human Right to Physical Integrity in the Biotechnical Age’ (2022) 9 J Law Biosci lsac032.

132
*Price* ECHR 2001-VII 153, 169, per Judge Greve.

133 Herring and Wall (n 11) 587.

134 See also, as discussed below: Jonathan Herring and Jesse Wall, “The Nature and Significance of the Right to Bodily Integrity” (2017) 76 Cambridge Law J 566.

135 McMahon (n 14).

136 There are debates on what is enhancement versus therapeutic use, these are outside the scope of this article.

137 For instance, if enhancement technologies, such as neural interface technologies enabling individuals to communicate with each other, were to develop, these could fundamentally alter how people with and without access to such technologies experience the world around them and communicate with others. Similarly, if in future, genetic screening, PGD, and genome sequencing became a more common part of human reproduction for those who can access it, these technologies could fundamentally change human reproduction, for a discussion on this, see: Hank Greely, *The End of Sex and the Future of Human Reproduction* (Harvard University Press 2018).

138 See discussion in: Francoise Baylis, *Altered Inheritance: CRISPR and the Ethics of Human Genome Editing* (Harvard University Press 2019).

139 <https://neuralink.com/> accessed 01 April 2025.

140 See discussion of this, in Antonio Regalado, ‘With Neuralink, Elon Musk Promises Human-to-Human Telepathy. Don’t Believe It’ (MIT Review, 22 April 2017) <https://www.technologyreview.com/2017/04/22/242999/with-neuralink-elon-musk-promises-human-to-human-telepathy-dont-believe-it/> accessed 01 April 2025.

141 TM Spranger, ‘Brain Patents as a Legal or Societal Challenge?’ (2023) 54 IIC 268.

142 Julian Savulescu, ‘New Breeds of Humans: The Moral Obligation to Enhance’ (2005) 1 Reprod Biomed Online 36; I Persson and J Savulescu, ‘The Duty to be Morally Enhanced ‘(2019) 38 Topoi 7.

143 David Cyranoski, Jorge L Contreras and Victoria T Carrington, ‘Intellectual Property and Assisted Reproductive Technology’ (2023) 41 Nat Biotechnol 41, 14.

144 ibid; Sonia M Suter, ‘In Vitro Gametogenesis: Just Another Way to Have a Baby?’ (2016) 3 J Law Biosci 87.

145 Hank T Greely, *The End of Sex and the Future of Human Reproduction* (Harvard University Press 2016) as cited by: Cyranoski and others (n 139).

146 art 5 Biotechnology Directive excludes the ‘human body, at the various stages of its formation and development…’ The Directive is supplementary interpretation for the EPC by the EPO (n 41). This approach is simliar in other jurisdictions, including the US context, see discussion in: Cyranoski et al, n 139, p. 17, which highlights that section 33(a) of the Leahy-Smith America Invents Act prohibits patents in the US context on 'claims directed to or encompassing a human organism'. They also note that the legislative history of the Act clarifies that patent claims on human embryos or fetuses are also prohibited in the US.

147 Case C-34/10 *Brustle v Greenpeace eV* [2011] E.C.R. I-9821; See EPO decision in: Wisconsin Alumni Research Foundation (WARF) (G002/06), Decision of the Enlarged Board of Appeal of 25 November 2008

148 See: Karen Walsh and Naomi Hawkins, ‘Expanding the Role of Morality and Public Policy in European Patent Law’ in Paul Torremans (ed), *Intellectual Property and Human Rights* (4th edn, Wolters Kluwer 2021).

149 Cyranoski, Contreras and Carrington (n 139), 14.

150 This states: ‘The rights conferred by a patent shall not extend to any of the following: (a) acts done privately and for non-commercial purposes.’

151 Natalie Rhodes and Remco van de Pas, ‘Mapping Buyer’s Clubs; What Role Do They Play in Achieving Equitable Access to Medicines?’ (2021) 17 Global Public Health 1842.

152 ibid.

153 In Australian context, see: Christopher Rudge and Narcyz Ghinea, ‘Promoting the Personal Importation of Therapeutic Goods: Recent Legislative Amendments to Advertising Regulations may Impact Consumer Access and Understanding’ (2023) 47 Aust Health Review 182.

154 Matthews and others (n 17) 2.3.3.

155 ibid. Lionel Bently and others, *Intellectual Property Law* (OUP 2022)

156 The requirements for CL are set out in: art 5(A) (2), Paris Agreement 1883, as amended; See discussion in: Esther van Zimmeren and Geertrui Van Overwalle, ‘A Paper Tiger? Compulsory License Regimes for Public Health in Europe’ 42 IIC 4; Aisling McMahon, ‘Patents, Access to Health and COVID-19: The Role of Compulsory and Government-use Licensing in Ireland’ (2020) 71 Northern Ireland LQ 331; Thambisetty and others (n 26).

157 ibid.

158 For an analysis of the compulsory licensing provisions under the Paris Agreement and limitations, see: McMahon (n 152); McMahon (n 17).

159 Oguanobi Hi, ‘Broadening the Conversation on the TRIPS Agreement: Access to Medicines Includes Addressing Access to Medical Devices’ (2018) 21 J World Intellect Prop 70; ‘t Hoen (n 108).

